# Serum 5′-Nucleotidase as a Novel Predictor of Adverse Clinical Outcomes after Percutaneous Coronary Intervention in Patients with Coronary Artery Disease

**DOI:** 10.31083/j.rcm2501017

**Published:** 2024-01-10

**Authors:** Mikereyi Aimaitijiang, Ting-Ting Wu, Ying-Ying Zheng, Xian-Geng Hou, Haitao Yang, Yi Yang, Xiang Xie

**Affiliations:** ^1^Department of Cardiology, First Affiliated Hospital of Xinjiang Medical University, 830054 Urumqi, Xinjiang, China

**Keywords:** 5′-nucleotidase (5′-NT), coronary artery disease (CAD), CD73, prognosis

## Abstract

**Background:**

The correlation between $5^{\prime }$-Nucleotidase ($5^{\prime }$-NT) and the clinical outcomes in coronary artery disease (CAD) patients following percutaneous coronary intervention (PCI) is not clear. This study aims to clarify this relationship.

**Methods:**

The PRACTICE study enrolled 15,250 patients between December 2016 and October 2021. After filtering out those without $5^{\prime }$-NT data, a total of 6555 patients were analyzed with a median follow-up of 24 months. Based on the receiver operating characteristic (ROC) curve analysis, a $5^{\prime }$-NT level of 5.57 U/L was selected as the optimal cutoff value. All research samples were divided into high-value (≥5.57 U/L, n = 2346) and low-value groups (<5.57 U/L, n = 4209). Key clinical outcomes included all-cause death (ACD), cardiovascular death (CD), major adverse cardiovascular events (MACE), and major adverse cardiovascular and cerebrovascular events (MACCE). After separating patients into high and low value groups, multivariate Cox regression analysis was used to correct for potential confounding variables. Finally, risk ratios and their 95% confidence intervals (CIs) were calculated.

**Results:**

During the follow-up period, 129 instances of ACD were recorded—49 cases (1.2%) in the low-value group and 80 cases (3.4%) in the high-value group. Similarly, 102 CDs occurred, including 42 low-value group cases (1.0%) and 60 high-value group cases (2.6%). A total of 363 MACE occurred, including 198 low-value group cases (4.7%) and 165 high-value group cases (7%). A total of 397 cases of MACCE occurred, including 227 low-value group cases (5.4%) and 170 high-value group cases (7.2%). As serum $5^{\prime }$-NT increased, the incidence of ACD, CD, MACE and MACCE increased. After multivariate Cox regression, high $5^{\prime }$-NT levels were linked with a 1.63-fold increase in ACD risk (hazard ratio [HR] = 2.630, 95% CI: [1.770–3.908], *p* < 0.001) when compared to low $5^{\prime }$-NT patients. Similarly, the risk of CD, MACE, and MACCE increased by 1.298-fold (HR = 2.298, 95% CI: [1.477–3.573], *p* < 0.001), 41% (HR = 1.410, 95% CI: [1.124–1.768], *p* = 0.003) and 30.5% (HR = 1.305, 95% CI: [1.049–1.623], *p* = 0.017), respectively.

**Conclusions:**

high serum $5^{\prime }$-NT levels were independently correlated with adverse clinical outcomes in CAD patients following PCI, affirming its potential as a prognostic indicator.

## 1. Introduction

In recent years, the incidence rate and mortality of coronary artery disease (CAD) have risen significantly, posing a growing threat to human health and safety [1], as well as increasing the economic burden on patients and society [2]. While percutaneous coronary intervention (PCI) technology advancements have revolutionized CAD management [3], some patients still experience adverse clinical outcomes [4]. Therefore, recent studies have focused on prognosis prediction and finding new post PCI markers. Some of the emerging PCI markers include apolipoprotein E (ApoE) [5], plasminogen activator inhibitor-1 (PAI-1) [6], and anti-apolipoprotein B-100 autoantibody (anti-apoB-100 Ab) [7]. Additionally, metrics including the neutrophil/lymphocyte ratio, monocyte/lymphocyte ratio, systemic inflammation response index, systemic immune-inflammation index offer insights into a patient’s inflammatory and immune status [8, 9, 10, 11, 12, 13, 14, 15, 16].

The role of ${\mathrm{ecto-5}}^{\prime }$-nucleotidase ($5^{\prime }$-NT) has recently garnered increasing attention. The $5^{\prime }$-NT enzyme was identified 60 years ago, and is found in skeletal muscle and heart tissue [17]. Also known as CD73 (Ecto-5’-nucleotidase—eN, also known as CD73, is a glycosylated protein bound to the outer surface of the plasma membrane by a glycosylphosphatidylinositol anchor (1) and co-localizes with detergent-resistant and glycolipid-rich membrane subdomains called lipid rafts [18]), $5^{\prime }$-NT is an intrinsic membrane glycoprotein widely present on mammalian cells [19, 20]. It can anchor and bind to glycosyl phosphatidylinositol located on the outer surface of the plasma membrane, allowing colocalization with detergent-resistant and lipid-rich membrane subdomains [18, 21, 22]. Functionally serum $5^{\prime }$-NT catalyzes $5^{\prime }$-nucleotides with the ability to hydrolyze the corresponding nucleosides [18]. Dixon and Purdon *et al*. [23] were the first to point out the clinical significance of serum $5^{\prime }$-NT levels for liver, gallbladder, and bone disease diagnostics. Although evidence has implicated $5^{\prime }$-NT with cardiovascular disease development, these results remain controversial [21, 22, 23, 24, 25, 26, 27, 28, 29, 30, 31, 32]. Some animal studies have suggested it exerts protective effect against ischemia, but other recent studies implicate $5^{\prime }$-NT in promoting atherosclerosis [33, 34, 35]. Thus, the relationship between $5^{\prime }$-NT and clinical outcomes in patients after PCI remains unclear. Given this uncertainty, we conducted a study involving 6555 patients with CAD after PCI to examine possible correlations between serum $5^{\prime }$-NT levels and clinical outcomes.

## 2. Methods

### 2.1 Study Design and Patients

The data used in this study came from a single-center prospective cohort study (PRACTICE). We analyzed the clinical records of CAD patients who underwent PCI at the First Affiliated Hospital of Xinjiang Medical University. The dataset spans from 2016-October 2021, including sex, age, smoking history, chronic disease history, laboratory results, and image examination data.

**Fig. 1.**
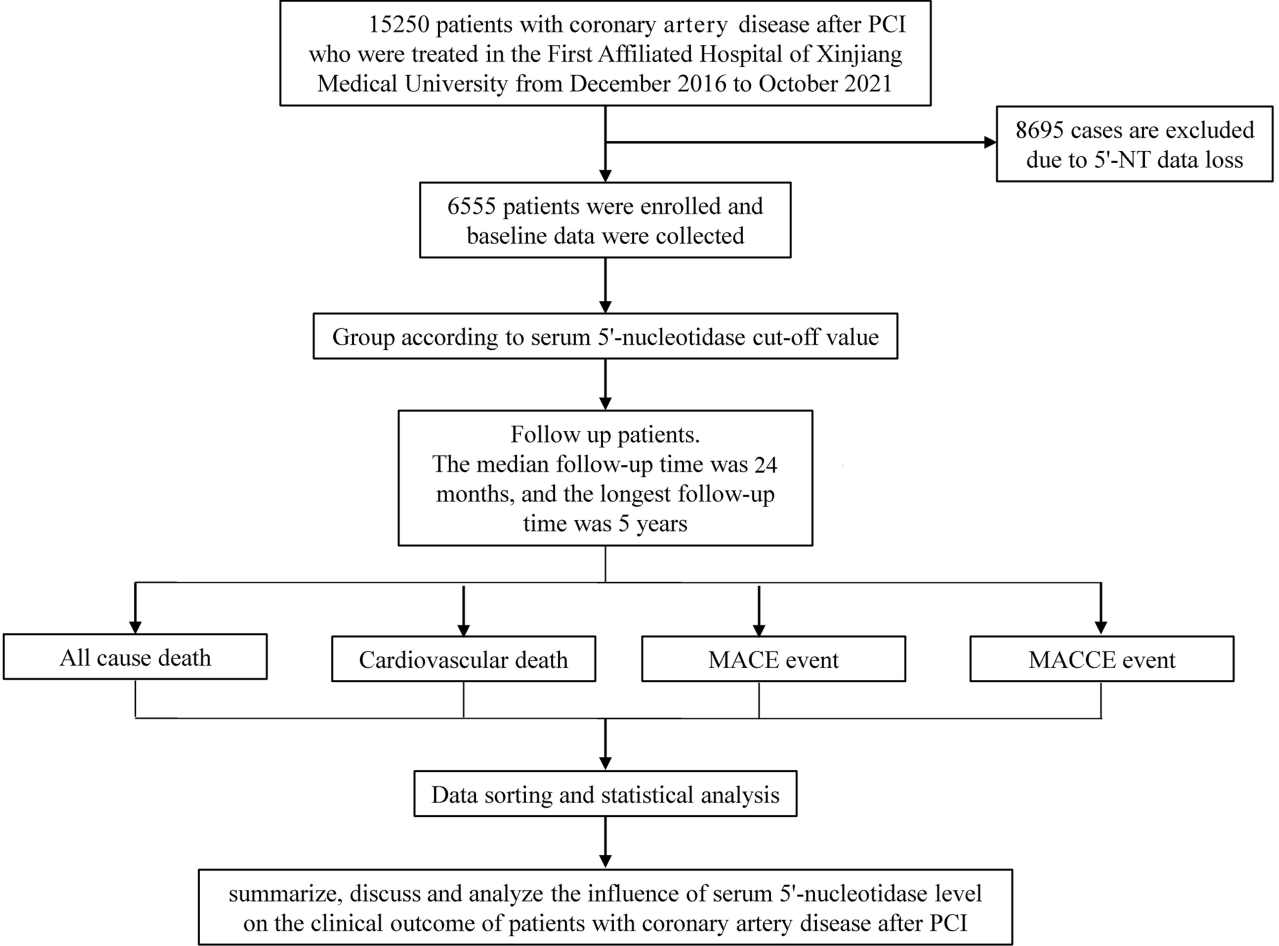
**Detailed inclusion and exclusion criteria**. PCI, percutaneous coronary intervention; $5^{\prime }$-NT, $5^{\prime }$-nucleotidase; MACE, major adverse cardiovascular events; MACCE, major adverse cardiovascular and cerebrovascular events.

The inclusion criteria consists of: (1) The results of severe coronary angiography, with at least one main coronary artery having ≥75% diameter stenosis. These vessels included the left main artery, left anterior descending branch, left bypass coronary artery, right coronary artery, and other important branches of the body (In the left main artery only, ≥50% diameter stenosis was the criterion for inclusion). (2) The implantation of at least 1 coronary stent by PCI. The exclusion criteria were as follows: (1) Complications from congenital heart disease or severe heart valve disease. (2) The presence of acute infectious diseases, malignant tumors, or hematological diseases. (3) Severe hepatic or renal insufficiency. (4) Incomplete clinical data. A total of 6555 patients met these criteria and were subsequently included in the study. Detailed inclusion and exclusion criteria are shown in Fig. 1.

### 2.2 Clinical and Demographic Characteristics Collection

This study required a comprehensive collection of clinical, laboratory, and imaging data from all patients. Blood was collected in the morning following an overnight fast. All laboratory examinations included in the study were conducted at the Physical Examination Center of the Affiliated Hospital of Xinjiang Medical University.

### 2.3 $5^{\prime }$-NT Detection

The enzyme rate method was used to measure serum $5^{\prime }$-NT. This test was also carried out in the medical laboratory center of the First Affiliated Hospital of Xinjiang Medical University. For the serum $5^{\prime }$-Nucleotide enzyme ($5^{\prime }$-NT) normal value, the enzyme rate method was conducted at 37 °C and 0–9 U/L. The colorimetric method used 2–17 U/L. Note: This experiment used the enzyme rate method; because plasma determination can cause turbidity of specimens and anticoagulants nucleated with metal ions will interfere with the activation of magnesium, it is generally run on serum. Hemolysis can make the result higher (The instrument used for this analysis: ultraviolet spectrophotometer).

#### Precautions before Drawing Blood

(1) The patients were told to not eat excessively greasy or high-protein foods the day before the blood test and to avoid excessive alcohol consumption. (2) After 8 p.m. the day before the physical examination, patients fasted for 12 hours to avoid affecting the test results. (3) During the blood draw, the patient was told to relax to avoid the constriction of blood vessels caused by fear, which can increase the difficulty of blood collection.

### 2.4 Follow-Up and Endpoints

The research conducted in this study was based on three kinds of follow-up data: the hospital’s inpatient system, outpatient electronic medical record system, and telephone interviews. Post-PCI patients were generally followed at 1 month, 3 months, 6 months, 1 year, 3 years and 5 years. The median follow-up time was 24 months, with the longest being 5 years. The primary endpoints we examined were death, all-cause death (ACD), and cardiogenic death (CD). The secondary endpoints included major adverse cardiovascular adverse events (MACE) and major adverse cardiovascular and cerebrovascular adverse events (MACCE). MACE was further subdivided into three types: cardiac death, recurrent myocardial infarction, and target vessel revascularization; MACCE additionally considered nonfatal stroke on top of MACE.

### 2.5 Statistical Analysis

This study applied SPSS 26.0 statistical analysis software (IBM Corp., Armonk, NY, USA) to process and analyze the obtained data. Continuous data (measurement data) are presented as the mean ± standard deviation, while categorical data (count data) are presented as percentages. The variables determined in the study were tested for normal distribution. The two-independent-sample *t* test or chi-squared test was applied to the intergroup data conforming normal distribution. The rank sum test was applied to the intergroup data not conforming to the normal distribution. Kaplan‒Meier curves were drawn to compare cumulative survival, followed by the log rank test. On this basis, a multifactor Cox regression analysis model was built to identify risk factors for the endpoints. The risk factors are presented in the form of hazard ratios (HRs) with their 95% confidence intervals (95% CIs). A value for *p* < 0.05 was considered significant.

## 3. Results

### 3.1 Baseline Data

At baseline, there were no significant differences in smoking, drinking, hypertension, urea or uric acid between the high- and ${\mathrm{low-5}}^{\prime }$-NT groups. However, the high value group did have a higher prevalence of diabetes, older age, a higher proportion of males, lower high-density lipoprotein cholesterol (HDL-C), higher total cholesterol (TC), and higher low-density lipoprotein cholesterol (LDL-C) (all *p <* 0.05) (Table 1).

**Table 1.** **Characteristics of our population**.

Parameters	Low serum 5′-NT (n = 4209)	High serum 5′-NT (n = 2346)	Chi-square or *t*	*p* value
Sex (male), n (%)	3061 (72.7)	1636 (69.7)	6.628	0.01
Smoking, n (%)	1668 (39.6)	916 (39.0)	0.215	0.643
Alcohol drinking, n (%)	1011 (24.0)	599 (25.5)	1.861	0.173
Hypertension, n (%)	3047 (72.8)	1655 (70.9)	2.555	0.11
Diabetes, n (%)	1717 (40.8)	1351 (57.6)	170.637	<0.001
Age, years	60.7 ± 11.3	60.1 ± 11.7	2.207	0.027
Urea, mmol/L	10.77 ± 38.33	10.45 ± 32.78	0.342	0.733
Uric acid, mmol/L	335.6 (279.6, 404.1)	358.0 (294.0, 439.0)	1.018	0.309
Total cholesterol, mmol/L	3.70 ± 1.04	3.95 ± 1.10	–8.648	<0.001
HDL-cholesterol, mmol/L	1.10 ± 0.30	1.07 ± 0.31	4.344	<0.001
LDL-cholesterol, mmol/L	2.34 ± 0.87	2.61 ± 0.93	–11.014	<0.001
CRP, mg/L	9.60 ± 25.10	19.12 ± 37.64	–6.762	<0.001
BNP, ng/L	1897.64 ± 3474.17	1294.33 ± 3120.22	0.502	0.619
AST, U/L	25.13 ± 29.34	51.84 ± 214.64	–5.997	<0.001
ALT, U/L	25.28 ± 26.75	47.48 ± 119.20	–8.895	<0.001
GGT, U/L	27.35 ± 25.25	63.55 ± 74.02	–22.954	<0.001
LVEF, %	60.44 ± 7.78	58.12 ± 9.203	10.144	<0.001
Single-vessel disease, n (%)	749 (17.8)	370 (15.8)	4.358	0.037
Multivessel disease, n (%)	3460 (82.2)	1976 (84.2)	4.358	0.037
LMCA, n (%)	302 (7.2)	213 (9.1)	7.545	0.006
RASi, n (%)	1745 (41.5)	1038 (44.2)	4.788	0.029
β-R(-), n (%)	2390 (58.4)	1367 (60.6)	2.737	0.098
Clopidogrel, n (%)	2099 (49.9)	1192 (50.8)	0.533	0.465
Ticagrelor, n (%)	2110 (50.1)	1154 (49.2)	0.533	0.465
Statins, n (%)	3955 (94.0)	2145 (91.4)	14.964	<0.001
Postoperative anticoagulation, n (%)	330 (7.8)	272 (11.6)	25.451	<0.001
SCAD, n (%)	1844 (43.8)	771 (32.9)	75.276	<0.001
ACS, n (%)	2365 (56.2)	1575 (67.1)	75.276	<0.001
Non-fatal myocardial infarction, n (%)	153 (3.6)	101 (4.3)	1.816	0.178
Stent thrombosis, n (%)	5 (0.1)	14 (0.6)	11.907	0.001

Values are mean ± SD or n (%). The *p* value indicates *p* for trend. HDL, high-density lipoprotein; LDL, low-density lipoprotein; $5^{\prime }$-NT, $5^{\prime }$-nucleotidase; CRP, C-reactive protein; BNP, brain natriuretic peptide; AST, aspartate transaminase; ALT, alanine transaminase; GGT, γ- Glutamine transpeptidase; LVEF, left ventricular ejection fraction; LMCA, left main coronary artery; RASi, renin-angiotensin-system inhibitor; β-R, β-receptor; SCAD, stable coronary artery disease; ACS, acute coronary syndrome.

Between the two $5^{\prime }$-NT groups, we compared C-reactive protein (CRP), brain natriuretic peptide (BNP), aspartate transaminase (AST), alanine transaminase (ALT), stable coronary artery disease (SCAD), acute coronary syndrome (ACS), r-glutamyltranspeptidase, left ventricular ejection fraction (LVEF), left main coronary artery (LMCA), renin-angiotensin-system inhibitor (RASi) usage β-blocker, single-vessel disease, multiple-vessel disease, clopidogrel, ticarello, statins, postoperative anticoagulation, nonfatal myocardial infarction, stent thrombosis and other aspects. The results of the empirical analysis indicate that there were no significant differences in BNP between the two groups of patients. In contrast, CRP, AST, ALT, and γ- Glutamine transpeptidase (GGT) were significantly elevated in the high-value group while LVEF was determined to be lower (all *p* < 0.05). There were no significant differences in R (-) [Receptor blocker], clopidogrel, ticarello, or nonfatal myocardial infarction. See Table 1 for details.

### 3.2 Grouping

Through the receiver operating characteristic curve analysis, we calculated Youden’s J statistic to identify the optimal cutoff point for $5^{\prime }$-NT levels. With both high specificity and sensitivity in mind, the optimal cutoff point was determined to be 5.57 U/L. Based on this threshold, patients were divided into a low-value group (<5.57 U/L, n = 4209) and a high-value group (≥5.57 U/L, n = 2346).

### 3.3 Incidence of Clinical Outcomes

**Fig. 2.**
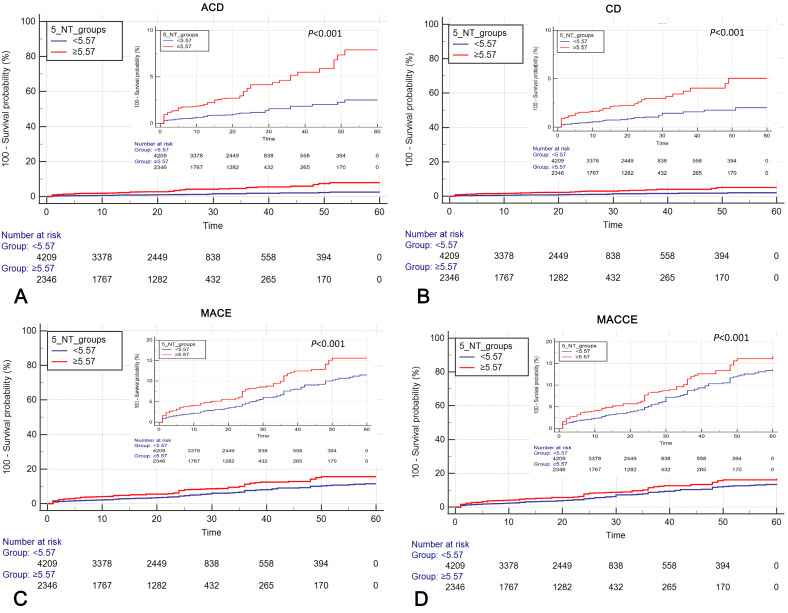
**Cumulative Kaplan‒Meier estimates of the time to first assessed occurrence of results during the 60-month follow-up**. (A) ACD. (B) CD. (C) MACE. (D) MACCE. $5^{\prime }$-NT, $5^{\prime }$-nucleotidase; ACD, all-cause death; CD, cardiogenic death; MACE, major adverse cardiovascular events; MACCE, major adverse cardiovascular and cerebrovascular events.

All 6555 patients were followed up for an average of 24 months and a maximum of 5 years. A total of 129 instances of ACD were reported during the follow-up period, including 49 cases (1.2%) in the low-value group and 80 cases (3.4%) in the high-value group. Similarly, 102 cardiovascular deaths (CDs) occurred, including 42 cases (1.0%) in the low-value group and 60 cases (2.6%) in the high-value group. MACE endpoints were noted in 363 patients, including 198 (4.7%) in the low-value group and 165 (7%) in the high-value group. A total of 397 patients reached the MACCE endpoint, including 227 (5.4%) in the low-value group and 170 (7.2%) in the high-value group. Statistical analysis revealed a significant increase in ACD, CD, MACE and MACCE incidences as serum $5^{\prime }$-NT levels rose (*p* < 0.05, Table 2). Kaplan‒Meier survival analysis confirmed positive correlations between elevated serum $5^{\prime }$-NT levels and the cumulative risk for ACD, CD, MACE, and MACCE (Fig. 2A–D).

**Table 2.** **Comparison of outcomes between the two groups**.

Outcomes	Low serum 5′-NT (n = 4209)	High serum 5′-NT (n = 2346)	Chi-square	*p* value
ACD, n (%)	49 (1.2)	80 (3.4)	39.384	<0.001
CD, n (%)	42 (1.0)	60 (2.6)	23.922	<0.001
MACE, n (%)	198 (4.7)	165 (7.0)	15.621	<0.001
MACCE, n (%)	227 (5.4)	170 (7.2)	9.092	0.003

The *p* value indicates *p* for trend. $5^{\prime }$-NT, $5^{\prime }$-nucleotidase; ACD, all-cause death; CD, cardiovascular death; MACE, major adverse cardiovascular events; MACCE, major adverse cardiovascular and cerebrovascular events.

### 3.4 Multivariate COX Regression Analysis

**Fig. 3.**
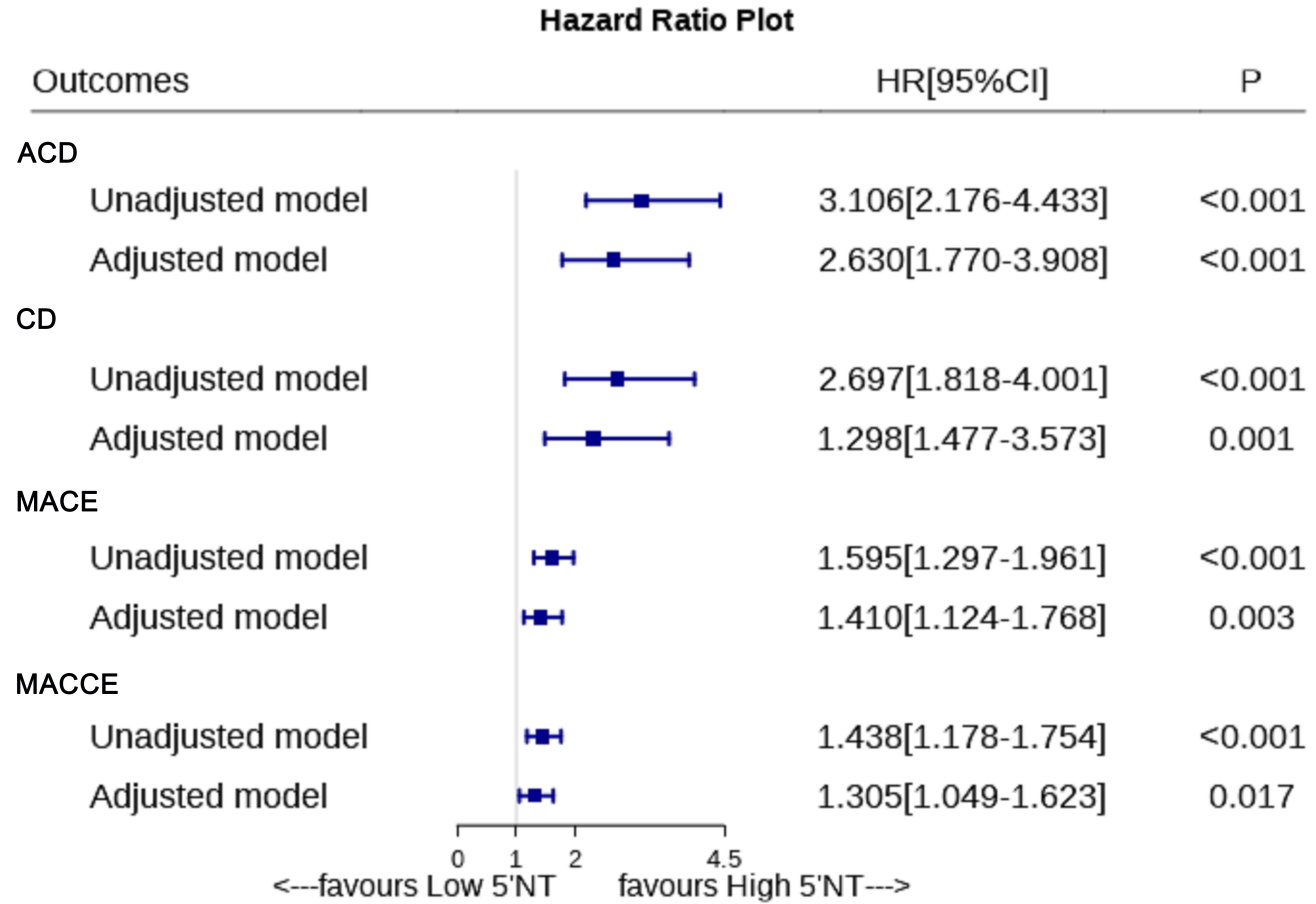
**Association of $5^{\prime }$-NT level with outcomes in univariate and multivariate models**. $5^{\prime }$-NT, $5^{\prime }$-nucleotidase; ACD, all-cause death; CD, cardiogenic death; MACE, major adverse cardiovascular events; MACCE, major adverse cardiovascular and cerebrovascular events; CI, confidence interval; HR, hazard ratio.

**Fig. 4.**
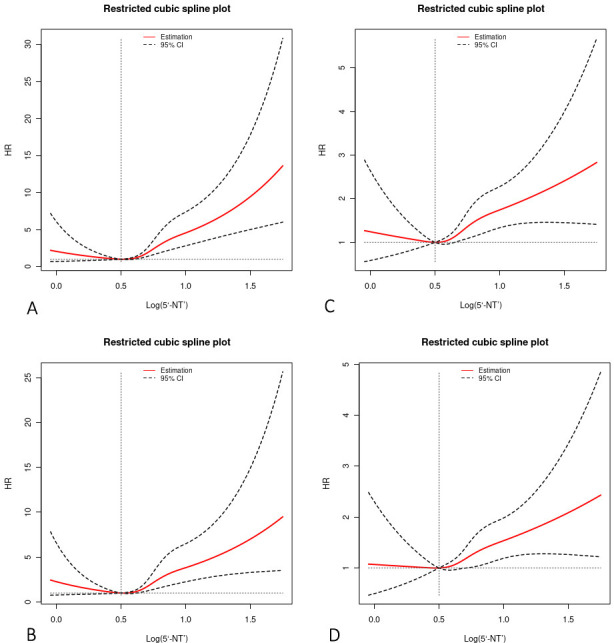
**Restricted cubic spline plots for ACD, CD, MACE, and MACCE by serum $5^{\prime }$-NT level after covariate adjustment**. Solid red central lines represent the estimated adjusted HRs, with black dotted lines denoting 95% confidence intervals. The horizontal dotted lines represent the HR of 1.0. The reference point was set at the lowest risk for outcomes in each plot (serum $5^{\prime }$-NT = 3.2 U/L). (A) Restricted cubic spline plot for ACD. (B) Restricted cubic spline plot for CD. (C) Restricted cubic spline plot for MACE. (D) Restricted cubic spline plot for MACCE. $5^{\prime }$-NT, $5^{\prime }$-nucleotidase; ACD, all-cause death; CD, cardiovascular death; MACE, major adverse cardiovascular events; MACCE, major adverse cardiovascular and cerebrovascular events; CI, confidence interval; HR, hazard ratio.

To mitigate potential biases, we employed a multivariate Cox regression model. After adjusting for variables including sex, age, diabetes history, HDL-C, LDL-C and other confounding factors, we assessed the impact of serum $5^{\prime }$-NT level on clinical outcomes (ACD, CD, MACE, and MACCE) in patients with CAD after PCI. The analysis revealed that patients with $5^{\prime }$-NT levels of <5.57 U/L had a 1.63-fold higher risk of experiencing ACD (hazard ratio [HR] = 2.630, 95% confidence interval [CI]: [1.770–3.908], *p* < 0.001) compared to those with $5^{\prime }$-NT levels ≥5.57 U/L. Similarly, the risks for CD, MACE, and MACCE were increased by 1.298-fold (HR = 2.298, 95% CI: [1.477–3.573] *p* < 0.001), 41% (HR = 1.410, 95% CI: [1.124–1.768], *p* = 0.003) and 30.5% (HR = 1.305, 95% CI: [1.049–1.623], *p* = 0.017), respectively (Fig. 3). Restricted cubic splines in the Cox regression analysis reflected a significant negative L-shaped relationship between outcomes and $5^{\prime }$-NT levels, further corroborating the link between elevated $5^{\prime }$-NT and adverse outcomes (Fig. 4A–D).

### 3.5 Subgroup Analyses

**Fig. 5.**
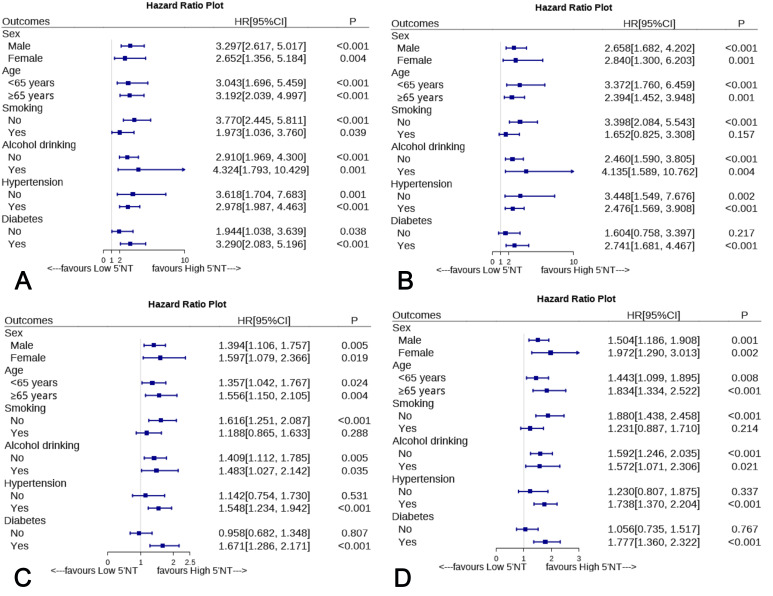
**Subgroup analyses of the relationship between serum $5^{\prime }$-NT and ACD (A), CD (B), MACE (C) and MACCE (D) according to age, sex, smoking, drinking, hypertension, and diabetes**. $5^{\prime }$-NT, $5^{\prime }$-nucleotidase; ACD, all-cause death; CD, cardiovascular death; MACE, major adverse cardiovascular events; MACCE, major adverse cardiovascular and cerebrovascular events; CI, confidence interval; HR, hazard ratio.

Fig. 5A–D reveal that age, sex, smoking, alcohol consumption, hypertension, or diabetes did significantly impact the relationship between elevated $5^{\prime }$-NT levels and ACD risk. However, elevated $5^{\prime }$-NT levels were not associated with CD, MACE, or MACCE in the smoking and nondiabetic subgroups. Furthermore, we also did not find elevated $5^{\prime }$-NT levels associated with MACE and MACCE in non-hypertensive patients.

## 4. Discussion

In this study, we propose that elevated $5^{\prime }$-NT levels serve as a prognostic risk marker, indicating poor long-term outcomes in patients with CAD after PCI. More specifically, the results of this empirical analysis establish a significant positive correlation between higher serum $5^{\prime }$-NT level and increased risks of long term mortality, as well as adverse cardiovascular and cerebrovascular events. As serum $5^{\prime }$-NT levels rise, the incidence of ACD, CD, MACE and MACCE also increase, providing new insights into the role of $5^{\prime }$-NT in CAD management.

While the role and mechanism of $5^{\prime }$-NT in cardiovascular diseases are controversial, animal studies have suggested it may have a cardioprotective function [21, 22, 23, 24, 25, 26, 27, 28, 29]. Specifically, increased $5^{\prime }$-NT activity and adenosine release have been shown to limit the scope of myocardial infarction in rodents [28]. Elevated $5^{\prime }$-NT may protect against CAD development by promoting adenosine production in ischemic myocardium [27, 28]. Adenosine, in turn, may protect the myocardium by activating K-ATP channels [21, 24, 25, 26, ]. Kitakaze *et al*. [28] also suggested that the short-term and high-dose administration of adenosine in dog hearts can activate $5^{\prime }$-NT, and thereby limit the scope of infarction, owing to adenosine’s cardioprotective properties mediated through $5^{\prime }$-NT [21, 22, 23, 24, 25, 26, 27, 28, 29].

Some studies challenge the purported cardioprotective role of $5^{\prime }$-NT. While *CD73+* regulatory T cells (Tregs) have been implicated in heart injury after ischemia/reperfusion, their role in repair after myocardial infarction (MI) remains unconfirmed [30, 31]. Contradicting previous findings, a recent study reported that inactivating the *CD73* gene did not impact the infarct size during ischemic preconditioning in mouse hearts either *in vitro* or *in vivo * [32]. This suggests that extracellular adenosine, generated by $5^{\prime }$-NT, may not contribute to the cardioprotective effects of adenosine during early ischemic preconditioning [32]. Thus, the role of $5^{\prime }$-NT in cardiovascular health is far from settled.

It has been reported [33] that* CD73* knockout led to arterial calcification, a ubiquitous pathological process of atherosclerosis [34]. This underscores the influence of *CD73* on the occurrence and progression of atherosclerosis. In a study using *ApoE*-/- mice, *CD73* inactivation inhibited the migration, proliferation and foam cell transformation of vascular smooth muscle cells (SMCs), thereby attenuating both AS and hyperlipidemia [35]. This led to the proposal that *CD73* is an important regulator in the development of atherosclerosis (AS). In their experiment,* CD73 siRNA* was used to downregulate *CD73* expression [35]. They mainly explain the role of* CD73* in the rupture of atherosclerotic plaques through the following mechanisms: First, deactivating *CD73* led to carotid artery ligation injuries, which affected parameters such as the neointimal area, the neointimal/medial thickness ratio, and the number of proliferative SMCs [35]. Second, the downregulation of *CD73* (at both the mRNA and protein levels) through siRNA significantly inhibited both the migration and growth of human umbilical artery smooth muscle cells (HUASMCs) [35]. Furthermore, the knockdown of *CD73* significantly reduced cyclin D1 levels, impacting the cell cycle [35]. This indicates that *CD73 *can inhibit the release and migration of inflammatory factors in HUASMCs, promoting their proliferation ability [35]. Third, administration of* CD73 siRNA *significantly reduced lipid accumulation, implying a role for *CD73* in lipid metabolism [35]. Fourth, *CD73* appears to promote plaque formation by increasing blood lipid levels, specifically triglycerides, TC, and plasma LDL-C [35]. The mechanism for these increases may be related to* CD73’s* regulatory impact on hepatic mRNA expression genes coding for hepatic lipase, peroxisome proliferator-activated receptor α, peroxisome proliferator-activated receptor γ, farnesyl diphosphate synthase, lecithin-cholesterol acyltransferase, and cytochrome p450 family 7 subfamily A member 1 in the liver [35]. Stenosis of the vascular lumen caused by abnormal lipid metabolism is the main cause of myocardial ischemia. Triglycerides, TC and LDL-C can be considered important risk factors for CAD [36].

Another study suggested impaired *CD73-*derived adenosine production contributes to the development of atherosclerosis in mice and humans, leading to calcification of human lower limb arteries [37]. When compared to the age-matched healthy control group, peripheral artery disease (PAD) patients had significantly higher *CD73* activity in the blood [37]. They suggest that the high* CD73* activity observed in the circulation of PAD patients appears to be a result of the shedding and loss of *CD73* expression in mature occlusive plaques [37]. Müller *et al*. [38, 39] proposed that signal-induced glycosylphosphatidylinositol-anchored *CD73* was upregulated by lysosomal degradation (LD)-mediated lipid synthesis in adipose tissue via diacylglycerol (DIG) transfer from the adipose body to adipocytes. They found that adipocytes release microbubbles containing *CD73*, which enter immature or small adipocytes through gaps or blood circulation and adhere to the surface of lipid droplets [38, 39]. Once attached, the enzymes facilitate the breakdown cAMP (cyclic adenosine monophosphate) on the lipid droplet surface [38, 39]. The decrease in cAMP levels impact lipid metabolism enzymes dependent on cAMP phosphorylation, such as hormone-sensitive lipase (HSL) and glycerol-3-phosphate acyltransferase (GPAT), thereby enhancing esterification, inhibiting lipolysis and promoting lipid synthesis [38, 39]. Furthermore, they described the release of specific transcripts and microRNAs induced by stimulation [40]. These molecules control lipid synthesis and lipid droplet biogenesis from primary and differentiated rat adipocytes to microbubbles containing Gce1 and *CD73* [40]. During their transfer and expression in small adipocytes, lipid synthesis is upregulated [40]. This suggests a potential mechanism for regulating lipid metabolism and adipocytes size, facilitated by microcapsules containing a specific set of GPI (glycolphosphatidylinositol)-anchored proteins and RNA [40]. In a separate study, Müller *et al*. [38] found that the esterification effects triggered by audiogenic stimulants could be nullified by depleting *CD73-*containing microvesicles secreted from adipocytes.

## 5. Conclusions

In our study, after adjusting for potential confounding variables, we found that the serum $5^{\prime }$-NT level could serve as an independent risk factor for long-term mortality in CAD patients after PCI. These findings suggest possible mechanisms explaining the role elevated serum $5^{\prime }$-NT levels play in the onset and progression of CAD.

This study has limitations, both subjective and objective in nature. First, it does not account for the influence of dietary habits, nutritional status, and other related factors in patients with CAD. Second, we did not measure serum $5^{\prime }$-NT activity in this study. Patients were only tested for $5^{\prime }$-NT levels on admission, without subsequent dynamic monitoring. Finally, this was a single-center cohort study. Although the sample was large, the patient population source was relatively narrow, undermining the study’s generalizability. Due to limited geographical representation of the population, unidentified confounding variables may affect the validity and reliability of the study findings. For a more comprehensive understanding, future research should aim for a larger, multicenter study design and include basic mechanistic experiments to better elucidate the relationship between serum $5^{\prime }$-NT levels and long-term outcomes in CAD patient following PCI. This would provide improved guidance for the comprehensive diagnosis and treatment of CAD.

## 6. Summary

This was the first study to investigate the relationship between elevated serum $5^{\prime }$-NT and long-term clinical outcomes in CAD patients following PCI treatment. The findings suggest that serum $5^{\prime }$-NT level can function as an independent prognostic marker for predicting adverse outcomes after PCI. Incorporating this biomarker into routine clinical practice could enhance decision-making in the treatment of coronary heart disease.

## Data Availability

Reasonable requests to access the data used in these analyses can be made to the first authors.
